# Rheumatoid arthritis in a patient with compound heterozygous variants in the *COL11A2* gene and progressive hearing loss: A case report

**DOI:** 10.1097/MD.0000000000028828

**Published:** 2022-02-18

**Authors:** Yumi Tsuchida, Yasuo Nagafuchi, Tomoko Uehara, Hisato Suzuki, Mamiko Yamada, Masanori Kono, Hiroaki Hatano, Hirofumi Shoda, Keishi Fujio, Kenjiro Kosaki

**Affiliations:** a Department of Allergy and Rheumatology, Graduate School of Medicine, The University of Tokyo, Tokyo, Japan; b Department of Functional Genomics and Immunological Diseases, Graduate School of Medicine, The University of Tokyo, Tokyo, Japan; c Center for Medical Genetics, Keio University School of Medicine, Tokyo, Japan; d Division of Clinical Genetics, Aichi Developmental Disability Center Hospital, Aichi, Japan.

**Keywords:** hearing loss, osteoarthritis, rheumatoid arthritis, type XI collagen

## Abstract

**Rationale:**

Collagen type XI alpha 2 chain is a component of type XI collagen and is expressed in various tissues including articular cartilage and tectorial membrane of the cochlea. Variants in the *COL11A2* gene, which encodes collagen type XI alpha 2 chain, has been reported to cause hearing loss and has been associated with osteoarthritis and ossification of the posterior longitudinal ligament of the spine. Despite the importance of type XI collagen in the joints, association of rheumatoid arthritis (RA) with *COL11A2* has not been reported.

**Patient concerns:**

The patient is a 60-year-old female, born to Japanese parents of no known consanguinity. She had progressive hearing loss since childhood. Her father also had progressive hearing loss before middle age. She developed joint pain in the knees and the hips in her forties. When she was 56, she developed polyarthritis. Rheumatoid factor and anti-CCP antibodies were positive.

**Diagnoses:**

She was diagnosed with osteoarthritis and RA. Whole exome analysis detected 2 rare variants, c.4201C>T, p.(Arg1401Trp) and c4265C>T, p.(Pro1422Leu), in the *COL11A2* gene (NM_080680.2). Whole genome analysis with a long insert size confirmed 2 variants that are in *trans*.

**Interventions and outcomes:**

She received a cochlear implant, which improved her hearing. She was treated with methotrexate, golimumab, tocilizumab, and upadacitinib with partial responses for her RA.

**Lessons:**

We herein report a patient with RA with compound heterozygous variants in the *COL11A2* gene. Autoantibodies against type XI collagen are detected in the sera of patients with RA, suggesting the possibility that type XI collagen may be involved in the pathogenesis of RA as an autoantigen. The hearing loss and osteoarthritis in this patient may be due to the compound heterozygous variants in the *COL11A2* gene, and the conformational changes induced by the variants may have changed the immunogenicity of type XI collagen, leading to the development of RA.

## 1 Introduction

*COL11A2* encodes collagen type XI alpha 2 chain, a component of type XI collagen.^[1]^ Type XI collagen forms heterotypic collagen fibrils with type II collagen and is expressed in various tissues including articular cartilage and tectorial membrane of the cochlea.^[2]^ *COL11A2* knockout mice exhibit small bodies, facial abnormalities, and deafness, as well as disorganized growth plate in long bones. In addition, chondrocytes in the knockout mice fail to align normally, and articular cartilages from these mice are thinner than those from wild-type mice.^[3]^ In humans, variants in *COL11A2* genes have been reported to cause autosomal dominant (DFNA13) and autosomal recessive (DFNB53) non-syndromic hearing loss^[4]^ and fibrochondrogenesis.^[5]^ Variants in the *COL11A2* also causes otospondylomegaepiphyseal dysplasia (OSMED), which is characterized by sensory hearing loss, disproportionately short limbs, enlarged epiphyses, vertebral abnormalities, and characteristic facies,^[6]^ demonstrating the importance of this gene in the development and homeostasis of the musculoskeletal system and the ear. Hearing loss in otospondylomegaepiphyseal dysplasia is not always evident at birth and may be progressive.^[7]^ Variations in the *COL11A2* gene have also been associated with osteoarthritis^[8]^ and ossification of the posterior longitudinal ligament of the spine.^[^9^,^^10^^]^

Despite the importance of type XI collagen in the homeostasis of joints, rheumatoid arthritis (RA) has not been described in association with variants in *COL11A2*, and we herein describe a patient with compound heterozygous variants in the *COL11A2* gene, who exhibited progressive sensory hearing loss since childhood and diagnosed with RA in her fifties.

## 2 Case report

A 60-year-old female patient had progressive hearing loss since early childhood. She was born to Japanese parents of no known consanguinity. Her father had progressive hearing loss before middle age, although the exact onset was not known. Her mother and 3 siblings did not have hearing loss. She had 3 children. One had been diagnosed with type I diabetes, and the other children were healthy. None of the children had hearing loss. There was no family history of joint disorders or systemic autoimmune diseases. She had never smoked.

**Figure 1:**
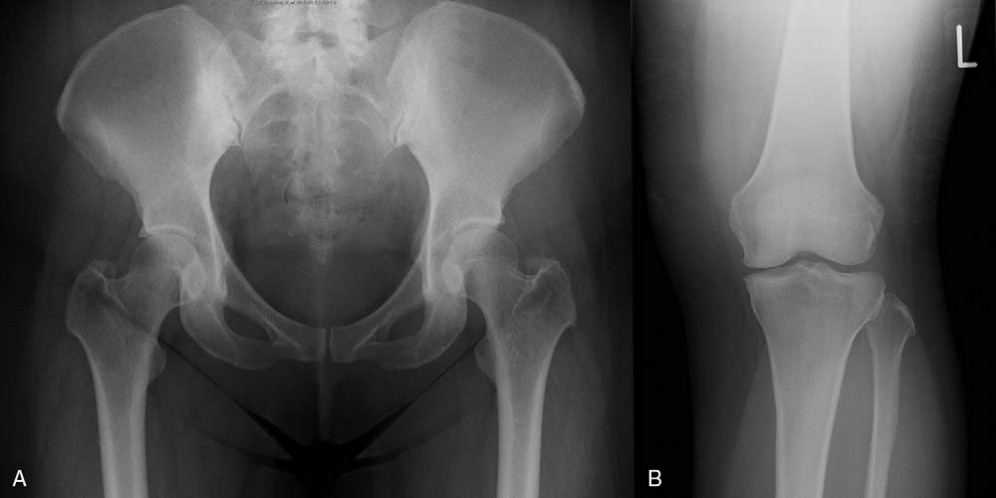
X-rays of the pelvis (A) and left knee (B) showing osteophytes and joint space narrowing, consistent with osteoarthritis.

**Figure 2:**
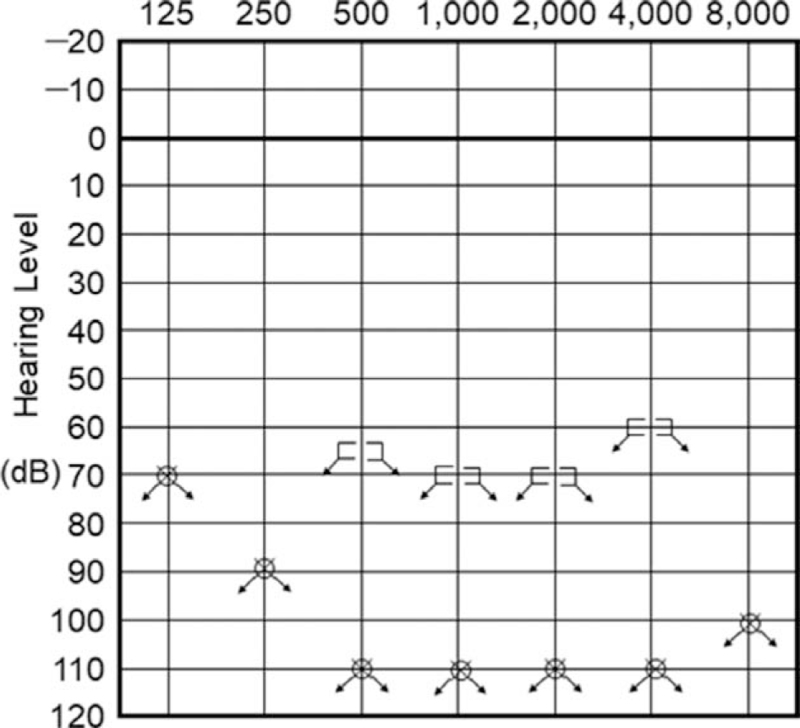
An audiogram performed at the age of 47.

The patient was referred to the otolaryngology department at our hospital when she was 46 years old for evaluation of hearing loss. Her hearing thresholds at this time was 115 dB in both ears. A computed tomography scan and an magnetic resonance imaging scan did not reveal any morphological abnormalities in the ears. She had no skeletal or facial abnormalities. Her C-reactive protein (CRP) was elevated at 2.65 mg/dL. Autoantibodies, including rheumatoid factor, anti-CCP antibody, anti-nuclear antibodies, and anti-neutrophil cytoplasmic antibodies were negative at this time. The patient had arthralgia in her hips and her knees, but there were no signs of synovitis, and X-rays were consistent with osteoarthritis (Fig. 1A and B). There were no signs of systemic autoimmune diseases or autoinflammatory diseases that could explain the elevation of inflammatory markers, such as systemic vasculitis. Because inflammatory markers were elevated, the possibility of autoimmune hearing loss was considered, and the patient was briefly treated with steroids. However, her hearing did not improve (Fig. 2). Two years later, she received a cochlear implant, which improved her hearing. Her inflammatory markers remained elevated, but the cause was not clear after extensive workup.

**Figure 3:**
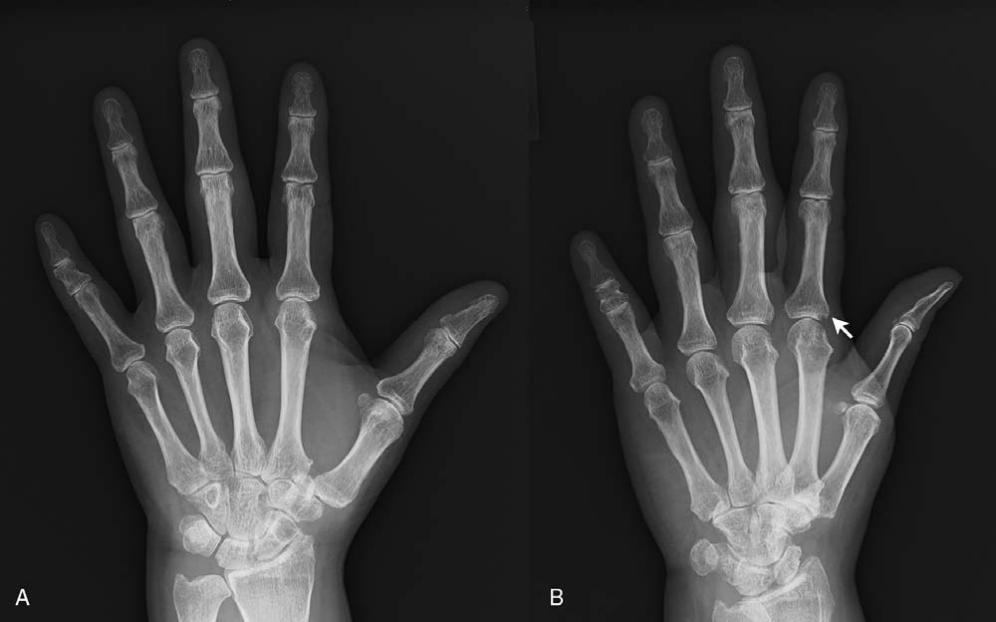
X-rays of the left hand (A: posteroanterior view and B: oblique view) a year after the diagnosis of RA. An erosion is seen in the 2^nd^ metacarpophalangeal joint, indicated with a white arrow. RA = rheumatoid arthritis.

When she was 56 years old, the patient noted morning stiffness and arthralgia in her wrist, metacarpophalangeal joints, elbows, and knees. She was referred to our department for evaluation. At this time, rheumatoid factor (33 IU/mL) and anti-CCP antibody (6.9 IU/mL) were positive. CRP was elevated at 14.0 mg/dL, and erythrocyte sedimentation rate was 91 mm/h. Ultrasound revealed synovitis in multiple joints, and although the clinical course was atypical and both rheumatoid factor and anti-CCP antibody titers were low, since there were no other diseases that could explain her synovitis, she was diagnosed with RA. She was treated with methotrexate. Due to nausea and elevation of liver enzymes, the dosage could not be increased to more than 8 mg/week, and 4 months later, she continued to have arthritis in multiple joints of her hands and the knees, and CRP remained elevated at 3.82 mg/dL. Golimumab was added at 50 mg/month and increased to 100 mg/month. Although her arthritis partially improved with the addition of golimumab, she continued to have arthritis in her hands, and her CRP remained elevated. A year later, her arthritis worsened, and CRP increased to 10.29 mg/dL. X-rays of the left hand showed erosions (Fig. 3), and the patient was switched to tocilizumab, an IL-6 inhibitor. After starting tocilizumab, her arthritis improved, and CRP normalized. However, 2 years later, she had another flare of her arthritis. Tocilizumab was switched to upadacitinib with improvement of her arthritis.

After obtaining written informed consent from the patient, genomic DNA was obtained from the patient. The patient provided written consent to participate in the study. This study was approved by the ethics committee at the University of Tokyo Hospital (G10109) and the ethics committee at the Keio University School of Medicine, Tokyo (A2015-106). Target regions were enriched with SureSelectXT Human All Exon V6 (Agilent Technologies), and whole exome sequencing was performed using Hiseq (Illumina). Nonsense variants, non-synonymous variants, splice acceptor site or donor site variants, and frameshift variants were filtered against dbSNP137,^[11]^ the 1000 Genomes Project (http://www.1000genomes.org/),^[12]^ ESP6500,^[13]^ the Japanese SNP dataset of 1208 normal individuals (Human Genetic Variation Browser: http://www.genome.med-kyoto-u.ac.jp/SnpDB),^[14]^ or a database of 3552 normal Japanese individuals (ToMMo: https://www.megabank.tohoku.ac.jp/english/).^[15]^

After the filtering process, there were 3 rare heterozygous variants in the *COL11A2* gene: NM_080680.2:c.74G>C, p.(Gly25Ala); NM_080680.2:c.4201C>T, p.(Arg1401Trp); and NM_080680.2:c4265C>T, p.(Pro1422Leu). We confirmed the results using Sanger sequencing. The variant frequencies were c.74G>C 0.0008; c.4201C>T 0.0005; and c4265C>T 0.0073 in ToMMo 4.7KJPN database (201909).^[15]^ The CADD Phred scores^[16]^ for each variants were c.74G>C 1.004; c.4201C>T 26.3; and c4265C>T 25.6. Among the 3 variants, the c.74G>C variant is likely benign because of low CADD Phred score of 1.004. As genomic DNA from the proband's parents were not available, to determine the relationship between the other 2 variants, c.4201C>T and c.4265C>T, a library of the patient's genomic DNA with a long insert size of 550 bp was prepared using TruSeq PCR free (Illumina), and a whole genome sequencing with a 10× coverage revealed that the 2 variants were in *trans*.

## 3 Discussion

Here, we report a patient with compound heterozygous c.4265C>T and c.4201C>T variants in the *COL11A2* gene who had progressive hearing loss and RA. The c.4265C>T variant has been reported to be associated with deafness in Japan.^[17]^ The other variant c.4201C>T has not been associated with any disease phenotype. We suspect that biallelic *COL11A2* variants in *trans* may have contributed to early development of osteoarthritis in addition to hearing loss.

The development of RA may be a coincidence; however, it is possible that the variants in the *COL11A2* gene may be involved in the development of RA since there are several lines of evidence that suggest a role for type XI collagen in the pathogenesis of RA. First, immunization of rats with type XI collagen causes arthritis similar to RA. Antibodies against type XI collagen, along with other collagen fibers, are detected in the sera of RA patients.^[18]^ Thus, type XI collagen could act as an autoantigen, leading to the production of autoantibodies, and in this patient, the conformational changes induced by the variants in the *COL11A2* gene may have changed the immunogenicity of type XI collagen, leading to the production of autoantibodies and arthritis. In addition, variants in the *COL11A2* gene have been reported to be associated with inflammatory disorders including Kawasaki disease,^[19]^ suggesting that abnormalities in type XI collagen may cause deregulation of the immune system. Furthermore, it has been suggested that the risk of RA is increased in patients with osteoarthritis, presumably due to increased inflammatory cytokines and citrullinated proteins in the joints of osteoarthritis patients,^[20]^ and osteoarthritis, caused by the variants in the *COL11A2* gene, may also have contributed to the development of seropositive, inflammatory arthritis in this patient.

In conclusion, we report a patient with progressive hearing loss and RA who had compound heterozygous non-synonymous variants in the *COL11A2* gene. A role for type XI collagen in the pathogenesis of RA has been suggested from animal studies and biochemical studies, and our data implicate a possible role for type XI collagen in the development of arthritis and autoimmunity from a genetic aspect.

## Author contributions

**Conceptualization:** Yumi Tsuchida, Yasuo Nagafuchi, Tomoko Uehara, Keishi Fujio, Kenjiro Kosaki.

**Data curation:** Yumi Tsuchida, Yasuo Nagafuchi, Masanori Kono, Hiroaki Hatano, Hirofumi Shoda.

**Formal analysis:** Tomoko Uehara, Hisato Suzuki, Mamiko Yamada, Kenjiro Kosaki.

**Project administration:** Keishi Fujio, Kenjiro Kosaki.

**Supervision:** Keishi Fujio, Kenjiro Kosaki.

**Writing – original draft:** Yumi Tsuchida, Yasuo Nagafuchi.

**Writing – review & editing:** Hirofumi Shoda, Keishi Fujio, Kenjiro Kosaki.
